# Configuration models as an urn problem

**DOI:** 10.1038/s41598-021-92519-y

**Published:** 2021-06-28

**Authors:** Giona Casiraghi, Vahan Nanumyan

**Affiliations:** ETH Zürich, Zürich, 8092 Switzerland

**Keywords:** Applied mathematics, Complex networks

## Abstract

A fundamental issue of network data science is the ability to discern observed features that can be expected at random from those beyond such expectations. Configuration models play a crucial role there, allowing us to compare observations against degree-corrected null-models. Nonetheless, existing formulations have limited large-scale data analysis applications either because they require expensive Monte-Carlo simulations or lack the required flexibility to model real-world systems. With the generalized hypergeometric ensemble, we address both problems. To achieve this, we map the configuration model to an urn problem, where edges are represented as balls in an appropriately constructed urn. Doing so, we obtain the generalized hypergeometric ensemble of random graphs: a random graph model reproducing and extending the properties of standard configuration models, with the critical advantage of a closed-form probability distribution.

## Introduction

Essential features of complex systems are inferred by studying the deviations of empirical observations from suitable stochastic models. Network models, in particular, have become the state of the art for complex systems analysis, where systems’ constituents are represented as vertices, and their interactions are viewed as edges and modelled by means of edge probabilities in a random graph. Such models are built to preserve certain properties of the observed network. How well the model describes other properties of the network distinguishes between randomly expected patterns and “interesting” ones.

The simplest of random graph models, known as the *G*(*n*, *p*), generates edges between a given number of vertices with a fixed probability $p \in (0,1]$^1^. In this model, the properties of vertices, such as their degrees, are homogeneously distributed, i.e., they have all the same expected value. However, most empirical graphs show heterogeneous, heavy tailed degree distributions^2–5^. Hence, random graph models able to incorporate *specified degree sequences* are of primal importance.

The most common family of models that fix degree sequences is known as the *configuration model* of random graphs. The comparison of an empirical graph with the corresponding configuration model allows quantifying which properties of the original graph can be ascribed to the degree sequence. The properties not explained by the degree sequence highlight the unique structure of the studied empirical graph. In particular, configuration models are invaluable for the macroscopic and the mesoscopic analysis of graphs. Its most notable applications, among others, are graph partitioning through modularity maximisation^6,7^ and quantifying degree correlations^8^.

The concept of a configuration model originates in the early works of Béla Bollobàs, who introduced the term ‘configuration’ to refer to the arrangement of edges in a model^9^. Since then, different configuration models preserving degrees have been proposed. In its standard formulation, the configuration model refers to the *uniform* sampling of a graph from the space of all graphs with a given degree sequence^10^. Such formulation thus fixes exactly the degree sequence and the number of edges in the model. Later, Molloy and Reed^11,12^ popularized the model, leading to the common reference in literature of *Molloy-Reed configuration model*.

Although being the most common version of the configuration model, that of Molloy and Reed is not the only possible one^13^. In particular, different types of configuration models arise if one allows for the existence of multi-edges or not, and of self-loops or not. Similarly, it is possible to distinguish between *vertex-labeled configuration models* and *stub-labeled configuration models*, as thoroughly discussed by^13^. Vertex-labeled graphs are those graphs for which there is a bijection between graph and adjacency matrix. This means that, in the case of a multi-edge graph, swapping the end-points of two multi-edges between the same pair of vertices leads back to the same graph. Stub-labeled graphs, on the other hand, are graphs for which swapping the end-points of two multi-edges between the same pair of vertices *does not lead back to the same graph*. For this reason, multiple distinct stub-labeled graphs may correspond to the same adjacency matrix. In this article, we focus only on vertex-labeled graphs, i.e., graphs that are completely identified by their adjacency matrix.

The standard configuration model has a crucial drawback: it can only be realized through a Monte-Carlo process, in the form of a repeated rewiring procedure. Each vertex is assigned a number of half-edges, or stubs, corresponding to their degree. A random realisation of the model is obtained by wiring pairs of stubs together uniformly at random. While implementing this procedure is relatively simple, uniformly sampling uncorrelated random realisation can be challenging^13^. This problem is exacerbated in the case of larger graphs and graphs with highly heterogeneous degree distributions. In particular, the mixing times, i.e., how many rewirings are needed before a sample from the algorithm is negligibly correlated with the starting graph, are poorly understood in the case of arbitrary degree distributions.

This limitation is a consequence of the hard constraints defining these models. Specifically, it stems from the choice of fixing exactly both the degree sequence of the model and the total number of edges, characteristics common in most variants of the standard configuration model. The only suitable approach to realize such models is that of Markov Chain Monte Carlo sampling (MCMC)^13^. The most popular algorithms rely on double edge swaps, also known as degree-preserving rewirings^14,15^, checkerboard swaps^16–18^, tetrads^19^, and alternating rectangles^20^, which randomize a given graph by simultaneously rewiring a pair of edges. This procedure had been already introduced in^21^ in 1891, and had been rediscovered many times later on^22–24^.

A solution to the lack of tractability and the poor scalability of MCMC based configuration models is relaxing the hard constraints characterising these models such that MCMC is not needed anymore. For this reason, there exist variants of the standard configuration model that relax the hard constraints of preserving exactly degree sequence and number of edges. Such models are often referred to as *soft-configuration models*. The soft-constrained graph model most closely related to the standard configuration model is the so-called *Chung–Lu model*^4, 25^. This model assumes that each edge (*i*, *j*) is drawn *independently* with probability $p_{ij}$ proportional to ${\mathbf {k}}^{\mathrm {out}}_i {\mathbf {k}}^{\mathrm {in}}_j/m$, where ${\mathbf {k}}^{\mathrm {out}}_i$ is the out-degree of vertex *i*. The Chung–Lu model can be seen as generalising the concept of the *G*(*n*, *p*) model^1^, such that not only the expected number of edges of the original graph is preserved, but also its degree distribution. In these terms, the standard configuration model instead can be seen as the degree-preserving counterpart of the *G*(*n*, *m*) model^10^, which generates all graphs with *n* vertices and *m* edges *uniformly*.

The Chung–Lu model is generally simpler to work with than the standard configuration model. However, it has the disadvantage of specifying not the actual values, but only the *expected* number of edges, and the *expected* degree sequence. This, among other things, means that it cannot specify the exact degree distribution^10^. Because of the essential role played by degree distributions, this issue has negatively affected the adoption of this model, in spite of its other advantages. Nevertheless, such an approach has found important applications, e.g., in the degree-corrected stochastic block model^26^. Note that the original Chung–Lu model was proposed for simple graphs^25^. However, because in this article we consider only multi-graphs, we will compare against its extension to multi-graphs introduced by^27^.

There exist further soft-configuration models formulations that fall into the family of exponential random graph models^10,13,28^. However, we will not discuss these here because they rely on MCMC as well^29^, and the mixing times of these chains are known to be very poor^30,31^.

In this article, we propose an analytically tractable model for random graphs with *given expected degree sequences* and a *fixed number of edges*. The model positions itself in between the standard configuration model, which fixes exactly the degree sequences, and the Chung–Lu model that preserves the number of edges in expectation and assumes that all edges are independent of each other. Thanks to this, it closely matches the properties of the standard configuration model, with the advantages given by an analytical formulation. The formulation of our model relies on mapping the process of drawing edges to a multivariate urn problem. Urn models are particularly useful to formalise complex sampling strategies, and have been already used to develop network null models in a few notable cases^32,33^. Thanks to the urn representation, our proposed model has, compared to other configuration models, the incomparable advantage of possibly incorporating *patterns that go beyond degree sequences*. In its simplest case, our model corresponds to a configuration model for directed or undirected multi-edge graphs, that fixes the values of vertex degrees in expectation instead of their exact values. In its general form, the model incorporates a parameter for each pair of vertices, which we call *edge propensities*. These parameters control the relative likelihood of drawing an individual edge between the respective pair of vertices, as opposed to any other pair. This is achieved by biasing the combinatorial edge drawing process. Any graph patterns can be modelled within this formulations, as long as they can be reduced to dyadic relations between pairs of vertices.

In the next section, we (1) provide formal definitions of the generalized hypergeometric ensemble of random graphs for the directed and undirected cases, and (2) investigate the properties of our model and how they compare against existing configuration models.

## Results

Let us consider a multi-graph ${\mathscr {G}}=(V,E)$, where $V$ is a set of $n$ vertices, and $E \subseteq V \times V$ is a multi-set of $m$ (directed or undirected) multi-edges. Specifically, a multi-edge *e* in the multi-set *E* is a tuple (*i*, *j*), where *i* and *j* are vertices in *V*. As there may be more than one multi-edge incident to the same pair of vertices *i*, *j*, we can refer to the multiplicity of the pair *i*, *j* as the number of multi-edges incident to it. We indicate with ${{\varvec{A}}}$ the adjacency matrix of the graph where entries $A_{ij}\in \mathbb {N}_{0}$ capture the number of multi-edges $(i,j)\in E$ that are incident to the pair *i*, *j*. In the case of undirected graphs, the adjacency matrix is symmetric, i.e., ${{\varvec{A}}} = {{\varvec{A}}}^T$, and the elements on its diagonal equal twice the multiplicity of the corresponding self-loops. Because we only deal with multi-graphs, in the rest of the article we will refer to multi-graphs simply as graphs, and to multi-edges simply as edges.

### Hypergeometric configuration model

**Figure 1:**
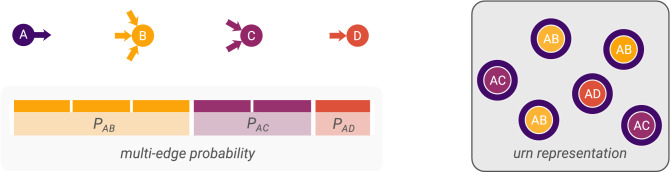
The configuration model represented (upper left) as a conventional edge rewiring process and (right) as an urn problem. In the former case, once the out-stub $(A,\cdot )$ has been sampled for rewiring, then one in-stub is sampled uniformly at random from those available, to draw a new multi-edge. If we represent each possible combination of an out-stub and an in-stub as a ball, we arrive at the urn problem without replacement. For the shown vertices, the odds of observing a multi-edge $(A,B)$ are three times higher than of observing a multi-edge between $(A,D)$ and $1.5$ times higher than of observing a multi-edge between $(A,C)$ in both model representations.

The concept underlying our random graph model is the same as that of the standard configuration model, which is to randomly shuffle the edges of a graph ${\mathscr {G}}$ while preserving vertex degrees. The standard configuration model generates edges one after another by sampling a vertex with an available out-stub (outwards half-edge) and a vertex with an available in-stub (inwards half-edge) until all stubs are consumed. Figure 1 illustrates one step of this process. The resulting random graphs all have exactly the same degree sequence as the original graph ${\mathscr {G}}$. If all pairs of available in- and out-stubs are equiprobable to be picked, so are the corresponding individual edges. Therefore, the probability of observing an edge between a given pair of vertices positively relates to the number of possible stub-pairings of the two vertices, which in turn is defined by the corresponding degrees of these. This probability depends only on the degrees of the two vertices and on the total number of edges in the graph.

The need to consequently sample two vertices at each step makes it cumbersome to formulate analytically the procedure described above. To overcome this challenge, we take an *edge-centric approach* of sampling $m$ edges from a certain larger multi-set, which we define below in Definition 1. As a consequence of this change of perspective, the model will preserve the *expected* degree sequences instead of the exact ones, resulting in a soft-configuration model. To this end, we introduce the definition of the *hypergeometric (soft-) configuration model*.

For each pair of vertices $i,j\in V$, we define the number $\Xi _{ij}$ of stub combinations that exist between vertices $i$ and $j$, which can be conveniently represented in matrix form:

#### Definition 1

(*Combinatorial matrix*) We define combinatorial matrix ${\varvec{\Xi }}\in \mathbb {N}^n\times \mathbb {N}^n$ for graph ${\mathscr {G}}$ as the matrix whose elements $\Xi _{ij}$ are defined as

1 $$\begin{aligned} \Xi _{ij} = {\mathbf {k}}^{\mathrm {out}}_i({\mathscr {G}}) {\mathbf {k}}^{\mathrm {in}}_j({\mathscr {G}}) \quad \mathrm {for~} i,j \in V, \end{aligned}$$

where ${\mathbf {k}}^{\mathrm {out}}({\mathscr {G}})$ and ${\mathbf {k}}^{\mathrm {in}}({\mathscr {G}})$ are the out-degree and in-degree sequences of the graph ${\mathscr {G}}$.

#### Hypergeometric configuration model for directed graphs

##### Definition 2

(*Directed hypergeometric configuration model*) Let ${\hat{{\mathbf {k}}}}^{\mathrm {in}},\,{\hat{{\mathbf {k}}}}^{\mathrm {out}}\in \mathbb {N}^n$ be in- and out-degree sequences and ${\hat{V}}$ a set of $n$ vertices. The hypergeometric configuration model (HypE) $X$ generated by $({\hat{V}},{\hat{{\mathbf {k}}}}^{\mathrm {in}},\,\hat{\mathbf {k}}^{\mathrm {out}})$ is the $n^2$-dimensional random vector $X$ defined on the probability space $(S,P)$ with sample space

2 $$\begin{aligned} S=\left\{ {\mathscr {G}}(V,E) \,\big \vert \, \left| E \right| = m \right\} ,\quad m = \sum _{i\in V}{\hat{\mathbf {k}}^{\mathrm {in}}_i}=\sum _{i\in V}{{\hat{{\mathbf {k}}}}^{\mathrm {out}}_i}, \end{aligned}$$

with some probability measure $P$, such that individual edges are equiprobable and the expected degree sequences of a realisation of $X$ are fixed:

3 $$\begin{aligned} {\mathbb {E}}_P\left[ {\mathbf {k}}^{\mathrm {in}}(X)\right] =\hat{\mathbf {k}}^{\mathrm {in}},\quad {\mathbb {E}}_P\left[ {\mathbf {k}}^{\mathrm {out}}(X)\right] ={\hat{{\mathbf {k}}}}^{\mathrm {out}}. \end{aligned}$$

This set-up allows to map the model to an *urn problem* and thus to arrive at a closed-form probability distribution function for it. Before doing so, we introduce the concept of *induced random model*.

##### Definition 3

(*Graph-induced random model*) We say that the graph ${\mathscr {G}}(V,E)$ induces the random model $X$, if the quantities $({\hat{V}},{\hat{{\mathbf {k}}}}^{\mathrm {in}},\hat{\mathbf {k}}^{\mathrm {out}})$ generating $X$ are computed from ${\mathscr {G}}$. i.e., ${\hat{V}}=V$, $\hat{\mathbf {k}}^{\mathrm {in}}={\mathbf {k}}^{\mathrm {in}}({\mathscr {G}})$, and $\hat{\mathbf {k}}^{\mathrm {out}}={\mathbf {k}}^{\mathrm {out}}({\mathscr {G}})$.

Under this assumption, we can formulate the following theorem for the distribution of the hypergeometric configuration model $X$. To keep the notation simple, we will not distinguish between the $n\times n$ adjacency matrix ${\varvec{A}}$ and the vector of length $n^{2}$ obtained by stacking it by row or column. Similarly, we do the same for all other related $n\times n$ matrices.

##### Theorem 1

(Distribution of the hypergeometric configuration model) *Let* ${\mathscr {G}}(V,E)$ *be a directed graph with* $n=\left| V \right|$ *vertices and* $m=\left| E \right|$ *edges. Let* ${\mathbf {k}}^{\mathrm {in}}({\mathscr {G}})\in \mathbb {N}^n$ *and* ${\mathbf {k}}^{\mathrm {out}}({\mathscr {G}})\in \mathbb {N}^n$ *be the vectors representing its in-degree and out-degree sequences. Let X be the hypergeometric configuration model induced by* ${\mathscr {G}}$ *defined as in Definition* 2. *If the probability measure* $P$ *depends only on the degree sequences in* ${\mathscr {G}}$ *and the total number of edges* $m=\left| E \right|$ *and all edges are equiprobable, then* $X$ *follows the multivariate hypergeometric distribution as in Eq.* (4).

* Let* ${\varvec{A}}\in \mathbb {N}_0^n\times \mathbb {N}_0^n$ *be an adjacency matrix and* ${\varvec{\Xi }}\in \mathbb {N}^n\times \mathbb {N}^n$ *be the combinatorial matrix induced by* ${\mathscr {G}}$ *. Then the hypergeometric configuration model* $X$ *is distributed as follows:*

4 $$\begin{aligned} \Pr (X={\mathscr {G}}) = \frac{\prod _{i,j\in V}\genfrac(){0.0pt}0{\Xi _{ij}}{A_{ij}}}{\genfrac(){0.0pt}0{M}{m}}, \end{aligned}$$

*where* $M=\sum _{i,j\in V}\Xi _{ij}$ *is the total number of stub combinations between all vertices.*

##### *Proof*

We want to sample $m$ edges connecting any of the in- and out-stub pairs such that all such edges are equiprobable. The total number of stubs combinations between any two vertices $i,j$ is given by $\Xi _{ij}={\mathbf {k}}^{\mathrm {out}}_i {\mathbf {k}}^{\mathrm {in}}_j$ (cf. Lemma 4 in the “SI” for a proof). We can hence define the random graph model as follows. We sample $m$ edges without replacement from the multi-set of size $\sum _{i,j\in V}\Xi _{ij}$ that combines all the possible stub pairs combinations $\Xi _{ij}$ between all pairs $i,j \in V$. We sample without replacement because we need to mimic the process of wiring stubs. Once a stub pair has been used, it cannot be sampled again. We can view this model as an *urn problem* where the edges to be sampled are represented by balls in an urn. By representing the edges connecting each different pair of vertices $(i,j)$ as balls of a unique colour, we obtain an urn with a total of $M=\sum _{i,j\in V}\Xi _{ij}$ balls of $n^{2}=\left| V\times V \right|$ different colours. With this, the sampling of a graph according to our model, corresponds to drawing exactly $m$ balls from this urn. Each adjacency matrix ${\varvec{A}}$ with $\sum _{i,j\in V} A_{ij}=m$ corresponds to one particular realisation drawn from this model. The probability of drawing exactly ${\varvec{A}}=\{A_{ij}\}_{i,j \in V}$ edges between each pair of vertices is given by the multivariate hypergeometric distribution. $\square$

From Theorem 1 we derive the following results, whose proofs follow directly from properties of the hypergeometric distribution.

##### Corollary 1

*For each pair of vertices* $i,j \in V$ *, the probability that* $X$ *has exactly* ${A}_{ij}$ *edges between* $i$ *and* $j$ *is given by the marginal distributions of the multivariate hypergeometric distribution, i.e.*

5 $$\begin{aligned} \Pr (X_{ij}={A}_{ij}) = \frac{\genfrac(){0.0pt}0{\Xi _{ij}}{{A}_{ij}}\genfrac(){0.0pt}0{M-\Xi _{ij}}{m-{A}_{ij}}}{\genfrac(){0.0pt}0{M}{m}}. \end{aligned}$$

*Note that Corollary* 1*does not imply that the different* $X_{ij}$ *are independent, as* $\prod \Pr (X_{ij}={A}_{ij})\ne \Pr (X={\mathscr {G}})$.

##### Corollary 2

*The expected in- and out-degree sequences of realisations of the directed hypergeometric configuration model* $X$ *correspond to the respective degree sequences of the graph* ${\mathscr {G}}$ *inducing* $X$.

##### *Proof*

For each pair of vertices $i,j$ we can calculate the expected number of edges $\mathbb {E}\left[ X_{ij} \right]$ as

6 $$\begin{aligned} \mathbb {E}\left[ X_{ij} \right] = m \frac{\Xi _{ij}}{M} \end{aligned}$$

Moreover, summing the rows and columns of matrix $\mathbb {E}\left[ X_{ij} \right]$ and assuming directed graphs with self-loops we can calculate the expected in- or out-degrees of all vertices as

7 $$\begin{aligned} \begin{aligned} \mathbb {E}\left[ k^{\mathrm {in}}_j(X) \right] =&\sum _{i \in V}{\mathbb {E}\left[ X_{ij} \right] } = m \frac{\sum _{i \in V} \hat{k}^{\mathrm {out}}_i \hat{k}^{\mathrm {in}}_j}{M} = \hat{k}^{\mathrm {in}}_j, \\ \mathbb {E}\left[ k^{\mathrm {out}}_i(X) \right] =&\sum _{j \in V}{\mathbb {E}\left[ X_{ij} \right] } = m \frac{\sum _{j \in V} \hat{k}^{\mathrm {out}}_i \hat{k}^{\mathrm {in}}_j}{M} = \hat{k}^{\mathrm {out}}_i. \end{aligned} \end{aligned}$$

Equation (7) confirms that the *expected* in- and out-degree sequence of realisations drawn from $X$ corresponds to the degree sequence of the given graph ${\mathscr {G}}$. $\square$

#### Hypergeometric configuration model for undirected graphs

So far, we have discussed the hypergeometric configuration model for directed graphs. Specifying the undirected case, on the other hand, requires some more efforts. The reason for this is that, under the assumptions described in the previous section, the undirected version of the hypergeometric configuration model is the degenerate case of its directed counterpart, where the direction of the edges is ignored. In particular, this implies that the random vector corresponding to the undirected model has half the dimensions of the directed one, because any undirected edge between two vertices $i$ and $j$ can either be generated as a directed edge $(i,j)$ or as a directed edge $(j,i)$. We provide a formal proof of this in Theorem 6, in the “SI”.

##### Definition 4

(*Undirected hypergeometric configuration model*) Let ${\hat{{\mathbf {k}}}}\in \mathbb {N}^n$ be a degree sequence and ${\hat{V}}$ a set of $n$ vertices. The hypergeometric configuration model $X$ generated by $({\hat{V}},{\hat{{\mathbf {k}}}})$ is the $(n^2+n)/2$-dimensional random vector $X$ defined on the probability space $(S,P)$, for the sample space

8 $$\begin{aligned} S=\left\{ {\mathscr {G}}(V,E) \,\big \vert \, \left| E \right| = m \right\} ,\quad 2m = \sum _{i\in V}{{\hat{{\mathbf {k}}}}_i}, \end{aligned}$$

with some probability measure $P$, such that individual edges are equiprobable and the expected degree sequence of a realisation of $X$ is fixed:

9 $$\begin{aligned} {\mathbb {E}}_P\left[ {\mathbf {k}}(X)\right] ={\hat{{\mathbf {k}}}}. \end{aligned}$$

At first sight, it would appear that the undirected hypergeometric configuration model can simply be obtained by restricting the directed model to $n(n+1)/2$ components corresponding to the upper-triangle and the diagonal of the adjacency matrix. That is, we would sample $m$ edges among pairs $i\le j \in V$ from the multi-set of stub combinations $\Xi _{ij}$ as defined in Eq. (1). However, the resulting model does not satisfy Definition 4, because its expected degree sequence does not equal the degree sequence that induced it. To show this, we follow the same reasoning adopted in the proof of Corollary 2. The expected degree $\mathbb {E}\left[ k_i(X) \right]$ of a vertex $i$ is equivalent to

$$\begin{aligned} \mathbb {E}\left[ k_i(X) \right] = \sum _{j \in V}{\mathbb {E}\left[ X_{ij} \right] } = m \frac{\sum _{i \in V} \hat{k}_i \hat{k}_j}{\sum _{i\le j\in V}\hat{k}_i \hat{k}_j} \ne \hat{k}_i. \end{aligned}$$

This approach is wrong because the total number of undirected stub combinations is larger than in the directed case. The reason for this is the symmetry in the process of wiring two stubs. Let $\hat{\mathbf {k}}_i$ be the degree of vertex $i$ and ${\hat{{\mathbf {k}}}}_j$ the degree of vertex $j$. To form a edge $(i,j)$, each one of the ${\hat{{\mathbf {k}}}}_i$ stubs of $i$ can be connected to all $\hat{\mathbf {k}}_j$ stubs of $j$, and vice versa, each of the $\hat{\mathbf {k}}_j$ stubs of $j$ can be connected to all ${\hat{{\mathbf {k}}}}_i$ stubs of $i$. Hence, the total number of combinations of stubs between vertices $i$ and $j$ equals to ${\hat{{\mathbf {k}}}}_i \hat{\mathbf {k}}_j + {\hat{{\mathbf {k}}}}_j {\hat{{\mathbf {k}}}}_i = 2 {\hat{{\mathbf {k}}}}_i {\hat{{\mathbf {k}}}}_j$.

We can now formulate the equivalent of Theorem 1 for the undirected case. The distribution underlying the undirected hypergeometric configuration model can then be computed analogously to Theorem 1.

##### Theorem 2

(Distribution of undirected hypergeometric configuration model) *Let* ${\mathscr {G}}(V,E)$ *be an undirected graph with* $n=\left| V \right|$ *vertices and* $m=\left| E \right|$ *edges. Let* ${\mathbf {k}}\in \mathbb {N}^n$ *be the vector representing its degree sequence. Let* $X$ *be the undirected hypergeometric configuration model induced by* ${\mathscr {G}}$ *defined as in Definition* 4. *If the probability distribution underlying* $X$ *depends only on the degree sequence of* ${\mathscr {G}}$ *and the total number of edges* $m$ *, and all edges are equiprobable, then* $X$ *follows the multivariate hypergeometric distribution given in Eq. * (10).

* Let* ${\varvec{A}} \in \mathbb {N}_0^n \times \mathbb {N}_0^n$ *be the symmetric adjacency matrix corresponding to an undirected graph, and* $\Xi _{ij} = {\mathbf {k}}_i {\mathbf {k}}_j$ *be the combinatorial matrix induced by* ${\mathscr {G}}$ *. Then the undirected hypergeometric configuration model* $X$ *is distributed as follows:*

10 $$\begin{aligned} \Pr (X={\mathscr {G}}) = \frac{\prod _{i<j\in V}\genfrac(){0.0pt}0{2\Xi _{ij}}{A_{ij}} \prod _{l\in V}\genfrac(){0.0pt}0{\Xi _{ll}}{A_{ll}/2}}{\genfrac(){0.0pt}0{M}{m}}, \end{aligned}$$

*where* $M=\sum _{i<j\in V} 2\Xi _{ij} + \sum _{l\in V} \Xi _{ll} = \sum _{i,j\in V} \Xi _{ij}$ *is the total number of undirected stub combinations between all pair of vertices.*

##### *Proof*

The proof follows the same reasoning of the proof of Theorem 1, accounting for the fact that the total number of stubs combinations is now given by

11 $$\begin{aligned} {\left\{ \begin{array}{ll} 2 \Xi _{ij} &{} \mathrm {if~} i\ne j, \\ \Xi _{ii} &{} \mathrm {if~} i=j.\\ \end{array}\right. } \end{aligned}$$

$$\square$$

The undirected version of Corollarys 1 and 2 are obtained by choosing the suitable probability distribution, given by Eq. (10). For completeness, we report them in the “SI”.

### Comparison with other configuration models

In the sections above, we have provided a parsimonious formulation of a soft-configuration model. Specifically, we have done so in terms of an *hypergeometric ensemble*. The ensemble provides a random graph model formulation for directed and undirected graphs alike, in which (1) the expected in- and out-degree sequences are fixed, and (2) edges between these vertices with fixed expected degrees are formed uniformly at random. More precisely, the probability for a particular pair of vertices to be connected by an edge is only influenced by combinatorial effects, and thus only depends on the degrees of the vertices and the total number of edges.

We can now discuss the differences of this hypergeometric ensemble with the standard hard-constrained model and Chung–Lu configuration model variants. In particular, we compare here (1) the sample spaces of the three models, and (2) the distributions characterising the models. In the following, we take the standard model as the reference point.

Sample spaces of the configuration models. For a given degree sequence ${\varvec{k}}$, the standard configuration model samples *uniformly at random* from the space $S_{{\varvec{k}}}$ of all graphs with given degree sequence ${\varvec{k}}$. It can be easily specified for both directed or undirected graphs. The Chung–Lu model is instead defined on the space *S* of *all* graphs. Differently, the hypergeometric ensemble is defined on the space $S_m$ consisting of all graphs with a given number of edges $m=\sum {\varvec{k}}$. Because $S_{{\varvec{k}}}\subseteq S_m\subseteq S$, the hypergeometric ensemble is closer to the standard configuration model than the Chung–Lu model. The sample space of the Chung–Lu model is much larger than that of the hypergeometric ensemble and, furthermore, this increases the variance of the distribution of degrees, as we outline in the next paragraph.

Distributions of the three models. In the common formulation followed by^10,13^, the standard configuration model assumes all graphs with a given degree sequence to be equiprobable. Differently from this, both the hypergeometric ensemble and Chung–Lu model define a non-uniform probability distribution over a larger set of graphs. However, there is a major difference between the two latter models. While the hypergeometric ensemble—similarly to the standard configuration model—does not assume the independence of edge probabilities, in the Chung–Lu model edges are independent. This results in a large variance for both the distribution of the number of edges in the graph and the distribution of the degree of a vertex. In the multi-graph version of the Chung–Lu model, the probability of observing a directed graph ${\mathscr {G}}$ follows the Poisson distribution^27^

12 $$\begin{aligned} \Pr (X_{CL}={\mathscr {G}}):=\prod _{ij} (A_{ij}!)^{-1}\left( \frac{{\mathbf {k}}^{\mathrm {out}}_i{\mathbf {k}}^{\mathrm {in}}_j}{m}\right) ^{A_{ij}} e^{-\frac{{\mathbf {k}}^{\mathrm {out}}_i{\mathbf {k}}^{\mathrm {in}}_j}{m}}. \end{aligned}$$

This means that the probability distribution of the out-degree ${\mathbf {k}}^{\mathrm {out}}_i$ follows a Poisson distribution too, with parameter

13 $$\begin{aligned} \lambda :=\sum _j{\frac{{\mathbf {k}}^{\mathrm {out}}_i{\mathbf {k}}^{\mathrm {in}}_j}{m}}={\mathbf {k}}^{\mathrm {out}}_i. \end{aligned}$$

The same holds for in-degrees. This result follows from basic probability theory as the degree is simply the sum of *n* independent Poisson random variables^10^. Similarly, the total number of edges *m* in the graph follows a Poisson distribution with parameter $\lambda =m$. Because the variance of a Poisson distribution with parameter $\lambda$ is $\lambda$, we see that the variance of the number of edges in model grows linearly with the size of the graph.

In the case of the hypergeometric ensemble, the probability to observe a directed graph ${\mathscr {G}}$ is given by the hypergeometric distribution in Eq. (4). Hence, the distribution of the out-degree ${\mathbf {k}}^{\mathrm {out}}_i$ follows a hypergeometric distribution as well, as noted in the following corollary to Theorem 1.

#### Corollary 3

(Distribution of degrees) *Let* ${\mathscr {G}}$ *be a graph and* ${\mathbf {k}}^{\mathrm {out}}_i$ *the out-degree of vertex* *i*. *Under the hypergeometric ensemble, the probability that* ${\mathbf {k}}^{\mathrm {out}}_i=k$ *follows the hypergeometric distribution*

14 $$\begin{aligned} \Pr (k):=\frac{\genfrac(){0.0pt}0{m\cdot {\mathbf {k}}^{\mathrm {out}}_i}{k}\genfrac(){0.0pt}0{M-m\cdot {\mathbf {k}}^{\mathrm {out}}_i}{m-k}}{\genfrac(){0.0pt}0{M}{m}}. \end{aligned}$$

This result follows from the properties of the hypergeometric distribution, in particular from the fact that generating an out-degree *k* for vertex *i* corresponds to sampling exactly *k* out-stubs for vertex *i* from the pool of all out-stubs available $\sum _j\Xi _{ij}=\sum _j{\mathbf {k}}^{\mathrm {out}}_i{\mathbf {k}}^{\mathrm {in}}_j=m\cdot {\mathbf {k}}^{\mathrm {out}}_i$. From the previous corollary, we recover the value for the variance of the distribution of the degree of a vertex which is equal to ${\mathbf {k}}^{\mathrm {out}}_i(m-{\mathbf {k}}^{\mathrm {out}}_i)(m+1)^{-1}$. Because this variance is always smaller than ${\mathbf {k}}^{\mathrm {out}}_i$, the hypergeometric ensemble (HypE) more closely reproduces the standard configuration model (CM) compared to the Chung–Lu model (CL). Table 1 summarizes the differences between the three models.

**Table 1:** Properties of the three configuration models CM, HypE, and CL, arranged by increasing variance in the corresponding distributions.

Model	CM	HypE	CL
Distribution	Uniform	Hypergeometric	Poisson
Variance of *m*	–	–	*m*
Variance of degrees	–	${\mathbf {k}}^{\mathrm {out}}_i\frac{(m-{\mathbf {k}}^{\mathrm {out}}_i)}{(m+1)}$	${\mathbf {k}}^{\mathrm {out}}_i$

Numerical comparison. To further highlight the properties of the hypergeometric ensemble and to show how it relates with the other two configuration models, we perform a simple numerical investigation. First, we choose a degree sequence as a starting point. For this task, we use an empirical degree sequence, obtained from the top 285 vertices of the Gentoo collaboration network, ordered by degree, (cf.^34^). The network shows a heavy-tailed degree distribution, with a total of 1366 edges. Having fixed the degree sequence, we generate 50,000 realisations from each model and report the distribution of basic graph properties. In Fig. 1 provided in the “SI”, we also plot the simulated distributions obtained for the number of edges *m*, the mean squared error (MSE) of the degree sequences against the true one, the degree centralisation, and the degree assortativity.

A qualitative comparison shows that, in general, the hypergeometric ensemble fares considerably more closely to the standard configuration model than the Chung–Lu model. This is a consequence of the properties highlighted in the previous paragraph. Such qualitative evaluation can be confirmed quantitatively by comparing the distributions of the different properties. The realisations from the hypergeometric ensembles have in fact (1) a significantly smaller MSE ($p<1e-16$ obtained from a one-sided Welch two sample t-test), i.e., the degree sequences of the generated graphs deviates less from the original degree sequence, and (2) a significantly smaller variance in the degree centralisation (p-value $p<1e-16$ obtained from a one-sided bootstrap-t test). All three models provide similar degree assortativity distributions (p-value $p>0.1$ obtained from two-sided Kolmogorov–Smirnov tests). In the “SI”, we provide the details about the statistical comparisons of the different models.

### Generalized hypergeometric ensemble of graphs

Configuration models sample edges uniformly at random. However, such uniform sampling of edges is generally not enough to describe empirical systems. In empirical graphs, edge probabilities do not only depend on the degree of vertices but also on other characteristics. Examples of such characteristics are vertex labels that lead to observable group structures, distances between vertices, similarities, etc. Below we discuss how such influences on the sampling probabilities of edges can be encoded in a random graph model by means of a dyadic property we call *edge propensity*.

**Figure 2:**
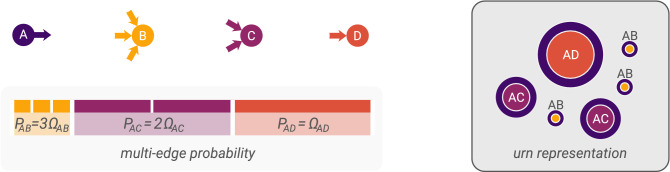
The effect of *edge propensities* on the configuration model. Differently from the standard configuration model, here the stubs are not sampled uniformly at random (cf. Fig. 1). Given an out-stub, each in-stub is characterized by a propensity $\Omega _{ij}$ of being chosen. As a result, the probability of wiring the out-stub $(A,\cdot )$ to the vertex $D$ is larger than that of $B$ due to a very large edge propensity $\Omega _{AD}$, even though vertex $B$ has three times more in-stubs than vertex $D$.

First, we introduce the concept of edge propensity. Given two dyads $(i,j)$ and $(k,l)\in V\times V$ where $\Xi _{ij}=\Xi _{kl}$, according to the hypergeometric configuration model the probabilities of sampling one edge between $(i,j)$ and $(k,l)$ are equal, leading to an odds-ratio of $1$ between the two pairs of vertices. Instead, we generalize the model in a way that fixing arbitrary odds-ratios is possible. That is, we specify edge propensities such that the ratio between them is the odds-ratio between the two corresponding vertex pairs of sampling a edge, all else being equal. We use edge propensities to bias the sampling probability of each edge, as illustrated in Fig. 2. This way, the probability of the number of edges between a pair of vertices depends on both the degrees of the vertices and their edge propensity.

We encode edge propensities in a matrix ${\varvec{\Omega }}$ defined as follows.

#### Definition 5

(*Propensity matrix*) Let ${\varvec{\Omega }}=(\Omega _{ij})_{i,j\in V}\in \mathbb {R}^{\left| V \right| \times \left| V \right| }$ be a $n\times n$ matrix where $n$ is the number of vertices. Let $(i,j)$ and $(k,l)\in V\times V$. Let $\omega _{ij,kl}$ the odds-ratio of sampling one edge between $(i,j)$ instead of $(k,l)$. The entries $\Omega _{ij}$ and $\Omega _{kl}$ of the propensity matrix ${\varvec{\Omega }}$ are then defined such that $\Omega _{ij}/\Omega _{kl}=\omega _{ij,kl}$. This implies that the propensity matrix ${\varvec{\Omega }}$ is defined up to a constant, as multiplying ${\varvec{\Omega }}$ with any constant preserves the specified odds-ratios.

Now, we define the generalized hypergeometric ensemble (gHyPE), a random graph model that combines degree-related combinatorial effects, i.e., the configuration model, and the newly introduced edge propensities. We do so by using propensities to *bias* the sampling process described above. In the urn model analogy, such biased sampling implies that the probability of drawing a certain number of balls of a given colour (i.e., edges between the corresponding pair of vertices) depends both on their number and their size, as illustrated in Fig. 2. The probability distribution resulting from such a biased sampling process is given by the multivariate * Wallenius’ non-central hypergeometric distribution*^35,36^.

#### Theorem 3

(Distribution of gHypEG) *Let* ${\mathscr {G}}(V,E)$ *be a directed graph with* $n=\left| V \right|$ *vertices and* $m=\left| E \right|$ *edges. Under the assumptions introduced above, the generalized hypergeometric ensemble of graphs (gHypEG)* $X$ *induced by* ${\mathscr {G}}$ *and a given propensity matrix* ${\varvec{\Omega }}$ *follows the* *multivariate Wallenius’ non-central hypergeometric distribution* *given in Eq.* (15).

*Let* ${\varvec{A}}\in \mathbb {N}^n\times \mathbb {N}^n$ *be the adjacency matrix associated with* ${\mathscr {G}}$ *and* ${\varvec{\Xi }}\in \mathbb {N}^n\times \mathbb {N}^n$ *be its* *combinatorial matrix* *defined in Eq.* (1). *Then the gHypEG defined by* ${\varvec{\Xi }}$ *and* ${\varvec{\Omega }}$, ${\mathscr {G}}$ *is distributed as follows:*

15 $$\begin{aligned} \Pr (X={\varvec{A}})=\left[ \prod _{i,j\in V}{\genfrac(){0.0pt}0{\Xi _{ij}}{A_{ij}}}\right] \int _{0}^{1}{\prod _{i,j\in V}{\left( 1-z^{\frac{\Omega _{ij}}{S_{{\varvec{\Omega }}} }}\right) ^{A_{ij}}}dz} \end{aligned}$$

*with*

16 $$\begin{aligned} S_{{\varvec{\Omega }}}= \sum _{i,j\in V} \Omega _{ij}(\Xi _{ij}-A_{ij}). \end{aligned}$$

The distribution describing the biased sampling from an urn is a generalisation of the multivariate hypergeometric distribution. Even though the probability distribution described by Eq. (15) is computed via numerical approximations^36^, it is possible to sample network realisations from it^37^. The R package BiasedUrn provides C++ implementations of such methods.

The proof of Theorem 3 follows from the fact that, when the sampling is performed without replacement with given relative odds, this sampling process corresponds to the multivariate Wallenius’ non-central hypergeometric distribution. Details of this derivation can be found in^35^ for the univariate case, and in^38,39^ for the multivariate case.

A thorough review of non-central hypergeometric distributions has been done in^37^.

The next two corollaries directly follow from properties of Wallenius’ non-central hypergeometric distribution.

#### Corollary 4

*For each pair of vertices* $i,j \in V$ *, the probability to draw exactly* $A_{ij}$ *edges between* *i* *and* *j* *is given by the marginal distributions of the multivariate Wallenius’ non-central hypergeometric distribution, i.e.,*

17 $$\begin{aligned} \begin{aligned} \Pr (X_{ij}=A_{ij}) = \genfrac(){0.0pt}0{\Xi _{ij}}{A_{ij}}\genfrac(){0.0pt}0{M-\Xi _{ij}}{m-A_{ij}}\cdot \int _{0}^{1} \left[ \left( 1 - z^{ \frac{\Omega _{ij}}{S_{{\varvec{\Omega }}}}} \right) ^{A_{ij}} \left( 1-z^{ \frac{\bar{\Omega }_{ij}}{S_{{\varvec{\Omega }}}}} \right) ^{m-A_{ij}} \right] dz \end{aligned} \end{aligned}$$

*where*

18 $$\begin{aligned} \bar{\Omega }_{ij} = \frac{\sum _{(l,m)\in \left( V\times V\right) \backslash (i,j)}{\Xi _{lm}\Omega _{lm}}}{(M-\Xi _{ij})}. \end{aligned}$$

#### Corollary 5

*The entries of the expected adjacency matrix* $\mathbb {E}\left[ X_{ij} \right]$ *can be obtained by solving the following system of equations:*

19 $$\begin{aligned} \left( 1-\frac{\mathbb {E}\left[ X_{11} \right] }{\Xi _{11}}\right) ^{\frac{1}{\Omega _{11}}} = \left( 1-\frac{\mathbb {E}\left[ X_{12} \right] }{\Xi _{12}}\right) ^{\frac{1}{\Omega _{12}}} = \ldots \end{aligned}$$

*with the constraint* $\sum _{i,j \in V} \mathbb {E}\left[ X_{ij} \right] = m$.

The undirected formulation of the gHypEG follows the same reasoning of Theorem 2, with the addition of a symmetric propensity matrix. That is, the distribution of the undirected generalized hypergeometric ensemble is hence given by a Wallenius’ distribution similar to Eq. (15), but corresponding to the upper triangular part of the matrices (i.e., for $i\le j$) in accordance with Eq. (11).

### Estimation of the propensity matrix

In this final section, we show how to define the propensity matrix ${\varvec{\Omega }}$ such that the expected graph $\mathbb {E}\left[ X \right]$ from the model $X$ defined by ${\varvec{\Omega }}$ coincides with an arbitrary graph $G$. By doing so, we create a random model *centered* around the inducing graph. The result is described in the following corollary, which follows from the properties of ’Wallenius’ non-central hypergeometric distribution.

#### Corollary 6

*Let* ${\mathscr {G}}(V,E)$ *be a graph with* $n=\left| V \right|$ *vertices and* ${\varvec{A}}$ *its adjacency matrix. Let* ${\varvec{\Omega }}$ *be a* $n\times n$ *propensity matrix, characterized by elements* $\Omega _{ij}$ *with* $i,j\in V$ *. Then,* ${\mathscr {G}}$ *coincides with the expectation* $\mathbb {E}\left[ X \right]$ *of the gHypEG* $X$ *induced by* ${\mathscr {G}}$ *and* ${\varvec{\Omega }}$ *if and only if the following relation holds.*

20 $$\begin{aligned} \forall c\in \mathbb {R}^-\quad \Omega _{ij} = \frac{1}{c}\log \left( 1 - A_{ij} / \Xi _{ij}\right) \;\forall i,j\in V, \end{aligned}$$

ıWhere ${\varvec{\Xi }}$ *is the combinatorial matrix associated with* $X$ *and* $\Xi _{ij}$ *its elements.*

#### *Proof*

Equation (20) follows directly from Corollary 4. In particular, when solving Eq. (19) for ${\varvec{\Omega }}$ with the assumption $\mathbb {E}\left[ X \right] = {\varvec{A}}$ we obtain the following system of $\left| V \right| ^2$ equations (in the case of a directed graph with self-loops) for $\left| V \right| ^2+1$ variables.

21 $$\begin{aligned} {\left\{ \begin{array}{ll} \left( 1-\frac{A_{11}}{\Xi _{11}}\right) ^{\frac{1}{\Omega _{11}}} &{}= C \\ \left( 1-\frac{A_{12}}{\Xi _{12}}\right) ^{\frac{1}{\Omega _{12}}} &{}= C \\ &{}\vdots \end{array}\right. } \end{aligned}$$

The solution of this system is Eq. (20). $\square$

A wide range of statistical patterns that go beyond degree effects can be encoded in the graph model by specifying the matrix ${\varvec{\Omega }}$ of edge propensities. The encoding and fitting techniques of such arbitrary propensity matrices are beyond the scope of this article, and will not be discussed here.

Finally, we highlight that the hypergeometric configuration model is a special case of the generalized hypergeometric ensemble. The hypergeometric configuration model described in Theorem 1 can be recovered from the generalized model by setting all entries in the propensity matrix to the same value. By doing so, the odds-ratio between the propensities for any pair of vertices is 1, and the edge sampling process is not biased. Thus, the probability distribution of the model reduces to a function of the degree sequences and the number of edges sampled. Theorem 7, in the “SI”, provides a formal proof for this result.

## Discussion

We have proposed a novel framework for the study of random multi-graphs in terms of an urn problem. By doing so, we have arrived at an analytically tractable formulation of the widely used configuration model of random graphs. It preserves degree sequences in expectation and the number of edges exactly. Our model relies on representing edges as balls in an urn containing the appropriate number of them, as explained in Definition 1. Sampling without replacement from such an urn generates random realizations from the model. Thanks to this parallel, we can identify the underlying probability distribution as the *multivariate hypergeometric distribution*. As we show, this formulation accurately reproduces the properties of standard configuration models, with the added benefit of a closed-form formulation.

We have further expanded such a configuration model to the *generalized hypergeometric ensemble*, a new class of models that can incorporate arbitrary dyadic biases into the probabilities of sampling edges. These biases, which we call edge propensities, control the relative likelihood of drawing an individual edge between the respective pair of vertices instead of any other pair. The proposed formulation allows incorporating into the model arbitrary graph patterns that can be reduced to dyadic relations. Again, in Theorem 3 we exploit the parallel to an urn problem to formalize the probability distribution underlying the generalized model: the *multivariate Wallenius’ non-central hypergeometric distribution*.

In^40^, we provide an open-source software implementation of the generalized hypergeometric ensemble within the R library ghypernet. The library is freely available for download on CRAN at https://cloud.r-project.org/package=ghypernet. The full library documentation can be found at https://ghyper.net.

## Supplementary information

Supplementary Information.

## References

[1] Erdős P, Rényi A (1959). On random graphs I. Publ. Math. Debrecen.

[2] Aiello, W., Chung, F. & Lu, L. A random graph model for massive graphs. In *Proceedings of the thirty-second annual ACM symposium on Theory of computing—STOC ’00*, 171–180. 10.1145/335305.335326 (ACM Press, 2000).

[3] Huberman BA, Adamic LA (1999). Growth dynamics of the World-Wide Web. Nature.

[4] Chung F, Lu L (2002). Connected components in random graphs with given expected degree sequences. Ann. Comb..

[5] Barabási A-L, Albert R (1999). Emergence of scaling in random networks. Science.

[6] Newman MEJ (2006). Modularity and community structure in networks. Proc. Natl. Acad. Sci..

[7] Radicchi F, Lancichinetti A, Ramasco JJ (2010). Combinatorial approach to modularity. Phys. Rev. E.

[8] Newman MEJ (2003). The structure and function of complex networks. SIAM Rev..

[9] Bollobás B (1980). A probabilistic proof of an asymptotic formula for the number of labelled regular graphs. Eur. J. Comb..

[10] Newman M (2018). Networks.

[11] Molloy M, Reed B (1995). A critical point for random graphs with a given degree sequence. Random Struct. Algorithms.

[12] Molloy M, Reed B (1998). The size of the giant component of a random graph with a given degree sequence. Comb. Probab. Comput..

[13] Fosdick BK, Larremore DB, Nishimura J, Ugander J (2018). Configuring random graph models with fixed degree sequences. SIAM Rev..

[14] Cafieri S, Hansen P, Liberti L (2010). Loops and multiple edges in modularity maximization of networks. Phys. Rev. E.

[15] Taylor R. Contrained switchings in graphs. In: McAvaney K.L. (eds) *Combinatorial Mathematics VIII. Lecture Notes in Mathematics*, vol. 884. Springer, Berlin, Heidelberg. 10.1007/BFb0091828 (1981).

[16] Stone L, Roberts A (1990). The checkerboard score and species distributions. Oecologia.

[17] Gotelli, N. J. & Graves, G. R. *Null Models in Ecology*. Washington, D.C.: Smithsonian Institution Press.(1996).

[18] Artzy-Randrup Y, Stone L (2005). Generating uniformly distributed random networks. Phys. Rev. E.

[19] Verhelst ND (2008). An efficient mcmc algorithm to sample binary matrices with fixed marginals. Psychometrika.

[20] Rao, A. R. *et al.* A Markov Chain Monte Carlo Method for Generating Random (0, 1)-Matrices with Given Marginals. Sankhyā: The Indian Journal of Statistics, Series A (1961–2002), 58(2), 225–242 (1996).

[21] Petersen J (1891). Die theorie der regulären graphs. Acta Math..

[22] Hakimi SL (1963). On realizability of a set of integers as degrees of the vertices of a linear graph. II. Uniqueness. J. Soc. Ind. Appl. Math..

[23] Newman MEJ (2003). Mixing patterns in networks. Phys. Rev. E.

[24] Bienstock D, Günlük O (1994). A degree sequence problem related to network design. Networks.

[25] Chung F, Lu L (2002). The average distances in random graphs with given expected degrees. Proc. Natl. Acad. Sci..

[26] Karrer B, Newman MEJ (2011). Stochastic blockmodels and community structure in networks. Phys. Rev. E.

[27] Norros I, Reittu H (2006). On a conditionally poissonian graph process. Adv. Appl. Probab..

[28] Snijders TA (2011). Statistical models for social networks. Annu. Rev. Sociol..

[29] Snijders TA (2002). Markov chain Monte Carlo estimation of exponential random graph models. J. Soc. Struct..

[30] Bhamidi, S., Bresler, G. & Sly, A. Mixing Time of Exponential Random Graphs. 49th Annual IEEE Symposium on Foundations of Computer Science. 803–812. 10.1109/FOCS.2008.75 (2008).

[31] Chatterjee S, Diaconis P (2013). Estimating and understanding exponential random graph models. Ann. Stat..

[32] Caldarelli G, Chessa A, Crimaldi I, Pammolli F (2013). Weighted networks as randomly reinforced urn processes. Phys. Rev. E.

[33] Marcaccioli R, Livan G (2019). A pólya urn approach to information filtering in complex networks. Nat. Commun..

[34] Zanetti, M. S., Scholtes, I., Tessone, C. J. & Schweitzer, F. The rise and fall of a central contributor: Dynamics of social organization and performance in the GENTOO community. 6th International Workshop on Cooperative and Human Aspects of Software Engineering (CHASE). pp. 49-56, 10.1109/CHASE.2013.6614731 (2013).

[35] Wallenius, K. T. Biased sampling: The noncentral hypergeometric probability distribution. Ph.d. thesis, Stanford University (1963).

[36] Fog A (2008). Calculation methods for Wallenius’ noncentral hypergeometric distribution. Commun. Stat. Simul. Comput..

[37] Fog A (2008). Sampling methods for Wallenius’ and fisher’s noncentral hypergeometric distributions. Commun. Stat. Simul. Comput..

[38] Chesson J (1978). Measuring preference in selective predation. Ecology.

[39] Chesson, J. A Non-Central Multivariate Hypergeometric Distribution Arising from Biased Sampling with Application to Selective Predation. *J. Appl. Probab.*, **13**(4), 795–797 (1976).

[40] Casiraghi, G. & Nanumyan, V. Ghypernet: Fit and simulate generalised hypergeometric ensembles of graphs. Version 1.0.1 10.5281/ZENODO.2555300 (2020).

