# Supplementary material for: Configuration models as an urn problem
Source: Sci Rep. 2021 Jun 28;11:13416. doi: 10.1038/s41598-021-92519-y (PMC8239003; doi:10.1038/s41598-021-92519-y)
Supplement: Supplementary file 1 — Supplementary Information. [file 41598_2021_92519_MOESM1_ESM.pdf]

Configuration models as an urn problem: the generalized hypergeometric ensemble of random graphs

Giona Casiraghi[1,*] **and Vahan Nanumyan**[1]

[1]Chair of Systems Design, ETH Zürich, Zürich, 8092, Switzerland
[*]gcasiraghi@ethz.ch

Supplementary Information (SI)

Additional Lemmas and Corollaries

Lemma 4 (Number of stub combinations)**.** *The combinatorial matrix $\Xi \in \mathbb{N}^n \times \mathbb{N}^n$ given in Definition 1 encodes the numbers of out-stub and in-stub combinations for each pair of vertices, given degree sequences $\mathbf{k}^{\text{out}}$ and $\mathbf{k}^{\text{in}}$.*

Proof. Let $\mathbf{k}_i^{\text{out}}$ be the out-degree of vertex i and $\mathbf{k}_j^{\text{in}}$ the in-degree of vertex j. The number of out-stubs of a vertex corresponds to its out-degree. Similarly, the number of in-stubs of a vertex corresponds to its in-degree. Each one of the $\mathbf{k}_i^{\text{out}}$ out-stubs can be connected to all $\mathbf{k}_j^{\text{in}}$ in-stubs. Hence, the total number of stub combinations between vertices i and j Ξ_{ij} is equal to $\mathbf{k}_i^{\text{out}}\mathbf{k}_j^{\text{in}}$. □

Corollary 2.1. *For each pair of vertices $i, j \in V$, the probability that X has exactly A_{ij} edges between i and j is given by the marginal distributions of the multivariate hypergeometric distribution in Eq. (10), i.e.,*

$$\Pr(X_{ij} = A_{ij}) = \begin{cases} \binom{2\Xi_{ij}}{A_{ij}} \binom{M - 2\Xi_{ij}}{m - A_{ij}} \binom{M}{m}^{-1} & \text{for } i \neq j, \\ \binom{\Xi_{ij}}{A_{ij}/2} \binom{M - \Xi_{ij}}{m - A_{ij}/2} \binom{M}{m}^{-1} & \text{for } i = j. \end{cases} \tag{1}$$

Corollary 2.2. *The* expected *degree sequence of realisations of X correspond to the respective degree sequence of the graph $\mathscr{G}$ inducing X.*

Proof. For each pair of vertices $i, j \in V$, the expected number of multi-edges $\mathbb{E}[X_{ij}]$ according to the hypergeometric distribution in Eq. (10) is expressed as

$$\mathbb{E}[X_{ij}] = 2m \frac{\Xi_{ij}}{M} \tag{2}$$

With this, we can write the expected degrees as

$$\mathbb{E}[k_j(X)] = \sum_{i \in V} \mathbb{E}[X_{ij}] = 2m \frac{\sum_{i \in V} \Xi_{ij}}{M} = 2m \frac{\sum_{i \in V} \hat{k}_i \hat{k}_j}{\sum_{i,j \in V} \hat{k}_i \hat{k}_j} = \hat{k}_j. \tag{3}$$

□

Numerical Comparison of CM, HypE, and CL

Implementation Here, we give some brief details about the the implementation of the three different models. The CM model, in particular, requires some caution. While the *stub-labeled* standard configuration model can be easily obtained by randomly rewiring edge pairs, this procedure give rises to a biased sampling of the *vertex-labeled multi-graphs*. Furthermore, such bias is not easily corrected a posteriori. Fosdick et al.[1] give a thorough investigation of the problem. The reason for this is the fact that rewiring two multi-edges incident to same pair of vertices give rises to two different stub-labeled graphs, but to the same vertex-labeled graph, thus inflating its sampling probability. Fosdick et al. provide a simple MCMC rejection sampling algorithm for the standard CM and vertex-labeled graphs (Algorithm 2[1]). The algorithm requires sampling two distinct edges $e_1 = (u, v)$ and $e_2 = (x, y)$ uniformly at random and then perform the rewiring $(u, v), (x, y) \to (u, y), (x, v)$. The graph obtained by rewiring is then accepted according to different probabilities based on the multiplicities of the different pairs of vertices involved in the swapping. In this way, the sample space of the CM is sampled uniformly at random. Two generate two uncorrelated samples from the algorithm, a large enough number of rewirings needs to be performed.

Sampling from the HypE or CL is simpler. In both cases, we can sample directly from the respective distributions. Because the CL assumes that edges incident to different pair of vertices are independent, a realisation from the CL is obtained by sampling from the univariate Poisson distribution for each vertices pair separately. Numerical sampling from the Poisson distribution can be performed in multiple ways[2]. In the following, we use the method proposed by[3], whose efficiency does not depend on the size of the mean of the distribution. This method is the standard algorithm provided in the `stats` library in `R`.

Finally, to sample realisation from the HypE, we simply need to sample a realisation from the multivariate hypergeometric distribution. As edges incident to different pairs of vertices are correlated, in this case we cannot perform independent sampling form univariate hypergeometric distributions. Gentle[2] highlights an efficient method to sample from the multivariate hypergeometric distribution exploiting its marginals. In the library `ghypernet`, the sampling routine `rghype` that we provide is based on this algorithm, as implemented in the `R` library `extraDistr`.

A Note About Performance The numerical performance of the three different models heavily depends on the implementation chosen. In general, we can note that the CL model is the most efficient of the three, as realisations for different vertex pairs can be generated in parallel. The main bottleneck of the CM is instead the mixing time of the MCMC algorithm. Mixing time results for non-simple graphs are poorly developed[1]. Moreover, rejection sampling in the vertex-labeled MCMC chain may increase mixing times for heterogenous degree sequences with many multi-edges and selfloops. Thus, many rewirings are needed in practice to ensure that realisations from the algorithm are uncorrelated. Mixing times increase further with the size of the network. The HypE positions itself between CM and CL. Because it requires sampling from a multivariate distribution, it is less efficient than CL. From numerical explorations perform using the implementations discussed above, we have observed that sampling from CL takes approximately 60% of the time needed to sample from HypE. This result does not vary significantly for increasing the number of vertices from 50 to 300 vertices. As discussed above, the performance of CM degrades quickly when increasing the number of vertices (increase in mixing time), and increasing average degrees (increase in number of rejections in the sampling algorithm). As the performance of CM depends in particular on its mixing time, and these are not well understood, we do not provide here a direct comparison of the sampling time of CM with the other models.

Numerical Comparison We provide the simulated distributions of some properties of the three configuration models realized from the same degree sequence. The empirical degree sequence corresponds to the degrees of the top 285 vertices of the Gentoo collaboration network, ordered by degree, (cf.[4]). It is characterized by a heavy-tailed degree distribution, with a total of 1366 edges as can be seen in Fig. 1a. The degree sequence is plotted in log-scale.

In Fig. 1, we compare the distributions of four different statistics for 50 000 realization obtained from the three different models CM, HypE, and CL. For each statistic, we create a density plot and superimpose the results of the different models. For the number of edges m, in Fig. 1b, we see that, as expected from theory, CM and HypE fix m exactly, while CL is characterized by a broad distribution. The mean squared error MSE of the simulated degree sequences against the true one are shown in Fig. 1c. It is evident how the MSE for HypE is both smaller on average and with a narrower distribution than CL. With respect to degree centralisation, in Fig. 1d it is possible to see that both CL and HypE are centered around the value of the original degree sequence, and that HypE has a narrower variance, with a distribution more peaked around the central value. Finally, the distributions of degree assortativities for the three models are indistinguishable, as shown in Fig. 1e.

To investigate quantitatively the qualitative insights described above, we perform a series of statistical tests. For the distributions of MSEs, we test:

1. the alternative hypothesis that HypE gives on average smaller MSEs than CL, using a one-sided Welch Two Sample t-test;
2. the alternative hypothesis that the distribution of the MSE for HypE is left-shifted compared to the CL (i.e., it is more probable that for two random realisations HypE has a smaller MSE compared to CL) by means of a one-sided Mann-Whitney non-parametric U test.

For both tests, we get a p-value $p < 1e-16$. For the degree centralisations, we perform a one-sided bootstrap-t test to compare the variances of the two distributions (also known as Normal-t test). We use $B = 50000$ bootstrap samples for the estimation of the test statistic. Also in this case, we get a small p-value $p < 1e-16$ testing the alternative hypothesis that the variance observed for the degree centralisation for HypE is smaller than that of CL. Finally, we compare the distributions of the degree assortativity obtained from the three models. We perform three two-sided Kolmogorov-Smirnov tests comparing the distributions in pairs. In all three cases, we get large p-values $p > .1$, signifying that the tests cannot distinguish between the three distributions, i.e., the null-hypotheses are not rejected.

Correspondence between directed and undirected models

We have formulated the hypergeometric configuration model for directed and undirected graphs independently of each other. We have motivated these models by the need of an analytically tractable analogy for the rewiring algorithm of the Molloy-Reed model[5]. This algorithm is the same in the directed and undirected case: select the first stub (outgoing, in the directed case), then select the second stub (incoming, in the directed case), create an edge by wiring these two stubs, and repeat the process until all the stubs are wired. Hence, we also show the correspondence between our directed and undirected formulations in this section.

We prove that the probability distribution of undirected graphs in the undirected hypergeometric configuration model given by Eq. (10) is a degenerate case of the directed model given by Eq. (4).

With the following definition, we provide a projection from $\mathbb{N}^{n^2}$ to $\mathbb{N}^{n(n+1)/2}$ that serves the purpose mapping a directed graph to its undirected equivalent, i.e., stripping the direction from its edges.

Definition 6 (Undirected projection)**.** Let $\mathscr{G}^{\updownarrow}(V,E^{\updownarrow})$ be a directed graph with adjacency matrix $\boldsymbol{A}^{\updownarrow}$. We define as *undirected projection* the map $\pi : \mathbb{N}^{n^2} \to \mathbb{N}^{n(n+1)/2}$ that maps $\mathscr{G}^{\updownarrow}$ to the undirected graph $\mathscr{G}(V,E)$ with adjacency matrix $\boldsymbol{A} = \boldsymbol{A}^{\updownarrow} + \boldsymbol{A}^{\updownarrow T}$. We indicate with $\mathscr{G}^{\updownarrow} \hookrightarrow \mathscr{G}$ the fact that $\mathscr{G} = \pi(\mathscr{G}^{\updownarrow})$.

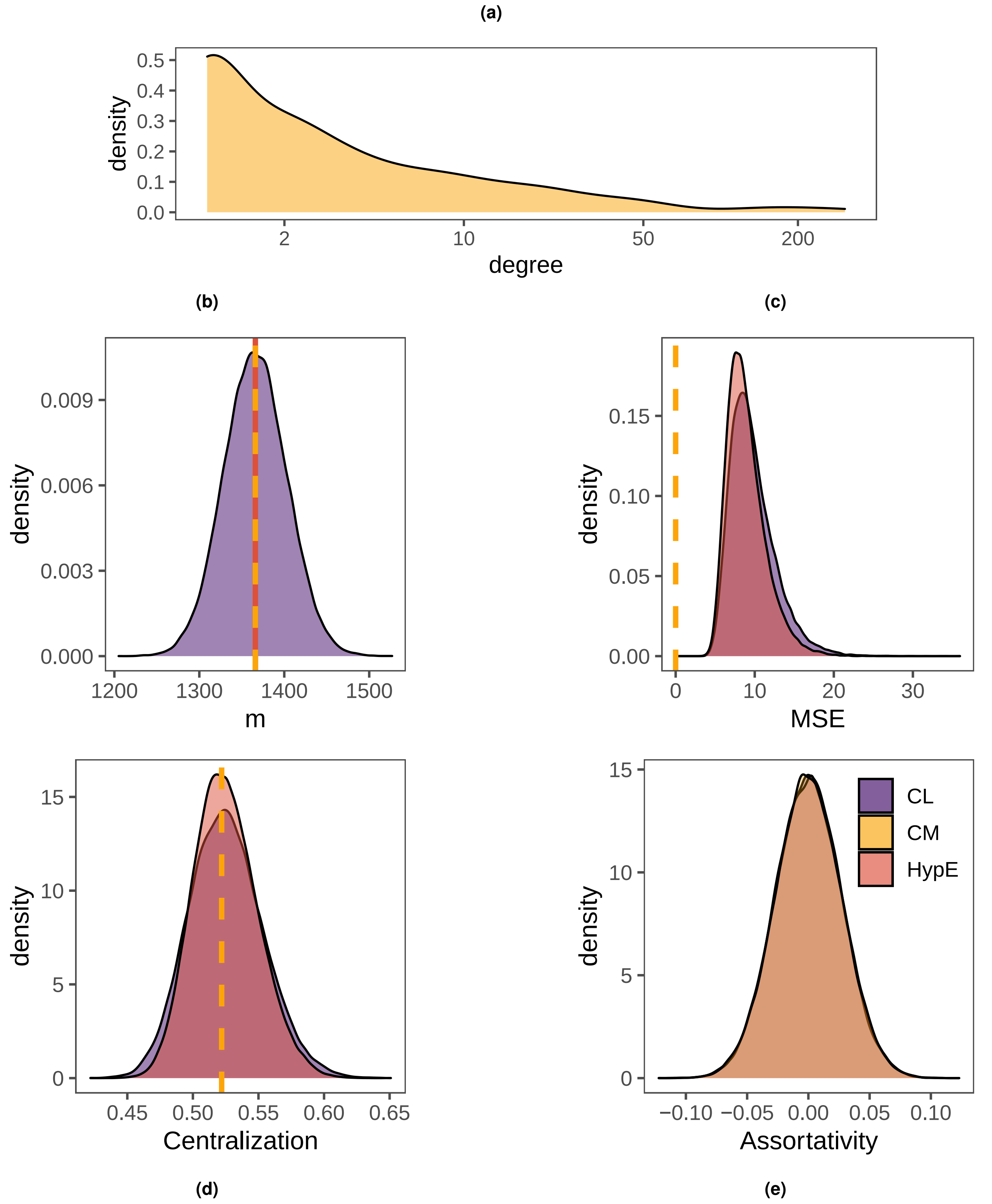

Figure 1. Numerical investigation of the three different configuration models: the standard configuration model (CM), the hypergeometric ensemble (HypE), and Chung-Lu model (CL). For each model, we generate 50 000 realisations, and we report the distribution of basic graph properties, i.e., **(b)** the number of edges m, **(c)** the mean squared error of the degree sequences, **(d)** the degree centralisation, and **(e)** the degree assortativity.

According to Definition 6, different directed graphs can be projected to the same undirected graph. At the same time, every undirected graph has at least one corresponding directed graph that can be projected to it, and for every directed graph there is at least one undirected graph to which it can be projected. These make the projection in Definition 6 surjective and not injective.

Similarly, we can define an undirected random graph model as the projection of a directed random graph model.

Definition 7 (Undirected projection of directed graph)**.** Let $X^{\updownarrow}$ be a directed random graph model. With abuse of notation, we use $X^{\updownarrow T}$ to refer to the transposition of the matrix representation of $X^{\updownarrow}$. We say that $X := X^{\updownarrow} + X^{\updownarrow T}$ is the undirected projection of $X^{\updownarrow}$ if $\forall \mathscr{G}^{\updownarrow}$ in the sample space of $X^{\updownarrow}$ exists a $\mathscr{G}$ in the sample space of X such that the undirected projection $\pi(\mathscr{G}^{\updownarrow})$ of $\mathscr{G}^{\updownarrow}$ is $\mathscr{G}$. Furthermore, for every undirected graph $\mathscr{G}$ in the sample space of X, $\exists \mathscr{G}^{\updownarrow}$ such that $\pi(\mathscr{G}^{\updownarrow}) = \mathscr{G}$. We indicate with $X^{\updownarrow} \hookrightarrow X$ the fact that X is the undirected projection of $X^{\updownarrow}$.

Note that according to Definition 6, the number of multi-edges m of $\mathscr{G}$ equals the number of multi-edges of any directed $\mathscr{G}^{\updownarrow}$ that projects to $\mathscr{G}$.

Finally, we need to relate the distribution underlying a directed random graph model to the distribution of its undirected projection. The following lemma serves this purpose.

Lemma 5 (Distribution of the directed projection)**.** *Let $\mathscr{G}$ be an undirected graph and X an undirected random graph model. Let $X^{\updownarrow}$ be a directed random graph model such that $X^{\updownarrow} \hookrightarrow X$. The probability distribution of X, $\Pr(X = \mathscr{G})$, is given as:*

$$\Pr(X = \mathscr{G}) = \sum_{\mathscr{G}^{\updownarrow} \in \pi^{-1}(\mathscr{G})} \Pr\left(X^{\updownarrow} = \mathscr{G}^{\updownarrow}\right), \tag{4}$$

where the set $\pi^{-1}(G) = \left\{\mathscr{G}^{\updownarrow} \,|\mathscr{G}^{\updownarrow} \hookrightarrow \mathscr{G}\right\}$ is the set of all directed graphs $\mathscr{G}^{\updownarrow}$ that map to $\mathscr{G}$.

Proof. Let $X^{\updownarrow}$ be a n^2-dimensional random vector formalising a directed random graph model, such that $X^{\updownarrow} \hookrightarrow X$. For simplicity, we index the elements of both random vectors as in the equivalent adjacency matrix notation. Let $X_{ij} = X_{ji}$ the ij-th element of X and $X^{\updownarrow}_{ij}, X^{\updownarrow}_{ji}$ the corresponding elements of $X^{\updownarrow}$.

According to Definition 7, X is the $n(n+1)/2$–dimensional random vector defined as $X^{\updownarrow} + X^{\updownarrow T}$, where its each element ij is defined as $X_{ij} = X^{\updownarrow}_{ij} + X^{\updownarrow T}_{ji}$. The probability distribution of X, $\Pr(X = \mathscr{G}) = f_X(\mathscr{G})$ can be specified in terms of the probability distribution $f_{X^{\updownarrow}}(\mathscr{G}^{\updownarrow}) = \Pr(X^{\updownarrow} = \mathscr{G}^{\updownarrow})$:

$$f_X(z) = f_X\left(\{z_{ij}\}_{ij}\right) = \sum \cdots \sum_{a_{ij}=0}^{z_{ij}} f_{X^{\updownarrow}}\left(\{z_{ij} - a_{ij}, a_{ij}\}_{ij,ji}\right) \tag{5}$$

The summation in Eq. (5) corresponds to the sum over the probabilities of all possible combinations of tuples $X^{\updownarrow}_{ij}, X^{\updownarrow}_{ji}$ which sum to A_{ij} for all indices ij. Hence, following Definition 6, this is equivalent to sum over all possible $\mathscr{G}^{\updownarrow} \hookrightarrow \mathscr{G}$. This proves the equivalence between Eq. (5) and Eq. (4) and thus, the lemma. □

We can proceed to show that the undirected version of the hypergeometric configuration model given in Theorem 1 is indeed equivalent to the model defined in Theorem 2. Theorem 6 stems from the fact that sampling an undirected edge between two vertices is equivalent to sampling a directed edge between the same pair of vertices in any of the two directions and then stripping its direction information. The distribution underlying the undirected hypergeometric configuration model can then be computed with the help of Lemma 5.

Theorem 6 (Correspondence between directed and undirected models)**.** *Let $\mathscr{G}$ be an undirected graph and X the undirected hypergeometric configuration model. Let $X^{\updownarrow}$ be the directed hypergeometric configuration model with combinatorial matrix with elements $\Xi_{ij} = \mathbf{k}_i \mathbf{k}_j$. The probability distribution of X is then given by Eq. (10).*

Proof. The distribution of $X^{\updownarrow}$ is given by the hypergeometric distribution in Eq. (4). The model $X^{\updownarrow}$ satisfies the conditions in Lemma 5 for the undirected hypergeometric configuration model X, because $X^{\updownarrow}$ maps to the undirected hypergeometric configuration model X in accordance with Definition 7. Hence, we write the probability distribution underlying X as the sum of

the probabilities of all corresponding directed graphs $\mathscr{G}^{\updownarrow}$ under the directed hypergeometric configuration model $X^{\updownarrow}$.

$$\Pr(X = \mathscr{G}) = \sum_{\mathscr{G}^{\updownarrow} \in \pi^{-1}(\mathscr{G})} \Pr\left(X^{\updownarrow} = \mathscr{G}^{\updownarrow}\right) \tag{6}$$

$$= \sum_{\mathscr{G}^{\updownarrow} \in \pi^{-1}(\mathscr{G})} \binom{M}{m}^{-1} \prod_{i,j \in V} \binom{\Xi_{ij}}{A^{\updownarrow}_{ij}} \tag{7}$$

$$= \sum_{\mathscr{G}^{\updownarrow} \in \pi^{-1}(\mathscr{G})} \binom{M}{m}^{-1} \prod_{l \in V} \binom{\Xi_{ll}}{A^{\updownarrow}_{ll}} \prod_{i<j \in V} \binom{\Xi_{ij}}{A^{\updownarrow}_{ij}} \binom{\Xi_{ij}}{A_{ij} - A^{\updownarrow}_{ij}} \tag{8}$$

$$= \sum \cdots \sum_{A^{\updownarrow}_{ij}=0}^{A_{ij}} \binom{M}{m}^{-1} \prod_{l \in V} \binom{\Xi_{ll}}{A^{\updownarrow}_{ll}} \prod_{i<j \in V} \binom{\Xi_{ij}}{A^{\updownarrow}_{ij}} \binom{\Xi_{ij}}{A_{ij} - A^{\updownarrow}_{ij}} \tag{9}$$

In Eq. (9) we have $n(n-1)/2$ summations for all $A^{\updownarrow}_{ij}$, $i < j$, which are the decomposition of the summation in Eq. (8). Then, we can swap the summations and multiplications in Eq. (9), which leads to

$$\Pr(\boldsymbol{A}) = \binom{M}{m}^{-1} \prod_{l \in V} \binom{\Xi_{ll}}{A^{\updownarrow}_{ll}} \prod_{i<j \in V} \sum_{A^{\updownarrow}_{ij}=0}^{A_{ij}} \binom{\Xi_{ij}}{A^{\updownarrow}_{ij}} \binom{\Xi_{ij}}{A_{ij} - A^{\updownarrow}_{ij}}. \tag{10}$$

From Vandermonde's identity, which states

$$\sum_{a=0}^{A_{ij}} \binom{\Xi_{ij}}{a} \binom{2\Xi_{ij} - \Xi_{ij}}{A_{ij} - a} = \binom{2\Xi_{ij}}{A_{ij}}, \tag{11}$$

and from the fact that $A^{\updownarrow}_{ii} = A_{ii}/2$, $\forall i \in V$, it follows that Eq. (10) is equivalent to Eq. (10). □

HypE as a special case of GHypE

Theorem 7 (Correspondence between gHypEG and hypergeometric configuration model)**.** *Let $\boldsymbol{\Omega} \equiv$ const. The corresponding gHypEG coincides with the hypergeometric configuration model in Eq.* (2) *induced by the same graph.*

Proof. For the special case of a uniform edge propensity matrix $\boldsymbol{\Omega} \equiv$ const, which corresponds to an unbiased sampling of edges, for the integral in Eq. (17) we have

$$\int_0^1 \left(1 - z^{\frac{1}{(M-m)}}\right)^m dz = \binom{M}{m}^{-1}. \tag{12}$$

Plugging this result in Eq. (15) we thus recover Eq. (4) for the unbiased case, i.e., where all edge propensities are identical. □

References

1. Fosdick, B. K., Larremore, D. B., Nishimura, J. & Ugander, J. Configuring random graph models with fixed degree sequences. *SIAM Rev.* **60**, 315–355 (2018).

2. Gentle, J. E. *Random Number Generation and Monte Carlo Methods*. Statistics and Computing (Springer-Verlag, New York, 2003).

3. Ahrens, J. H. & Dieter, U. Computer generation of poisson deviates from modified normal distributions. *ACM Transactions on Math. Softw. (TOMS)* **8**, 163–179 (1982).

4. Zanetti, M. S., Scholtes, I., Tessone, C. J. & Schweitzer, F. The Rise and Fall of a Central Contributor: Dynamics of Social Organization and Performance in the Gentoo Community. In *Chase*, 1–21 (2013).

5. Molloy, M. & Reed, B. A critical point for random graphs with a given degree sequence. *Random Struct. & Algorithms* **6**, 161–180, DOI: 10.1002/rsa.3240060204 (1995).
